# Transcriptomic response of *Sinorhizobium meliloti* to the predatory attack of *Myxococcus xanthus*

**DOI:** 10.3389/fmicb.2023.1213659

**Published:** 2023-06-19

**Authors:** María José Soto, Juana Pérez, José Muñoz-Dorado, Francisco Javier Contreras-Moreno, Aurelio Moraleda-Muñoz

**Affiliations:** ^1^Departamento de Biotecnología y Protección Ambiental, Estación Experimental del Zaidín, Consejo Superior de Investigaciones Científicas, Granada, Spain; ^2^Departamento de Microbiología, Facultad de Ciencias, Universidad de Granada, Granada, Spain

**Keywords:** bacterial predation, myxobacteria, *Sinorhizobium meliloti*, defensome, bacterial interactions

## Abstract

Bacterial predation impacts microbial community structures, which can have both positive and negative effects on plant and animal health and on environmental sustainability. *Myxococcus xanthus* is an epibiotic soil predator with a broad range of prey, including *Sinorhizobium meliloti*, which establishes nitrogen-fixing symbiosis with legumes. During the *M. xanthus*-*S. meliloti* interaction, the predator must adapt its transcriptome to kill and lyse the target (predatosome), and the prey must orchestrate a transcriptional response (defensome) to protect itself against the biotic stress caused by the predatory attack. Here, we describe the transcriptional changes taking place in *S. meliloti* in response to myxobacterial predation. The results indicate that the predator induces massive changes in the prey transcriptome with up-regulation of protein synthesis and secretion, energy generation, and fatty acid (FA) synthesis, while down-regulating genes required for FA degradation and carbohydrate transport and metabolism. The reconstruction of up-regulated pathways suggests that *S. meliloti* modifies the cell envelop by increasing the production of different surface polysaccharides (SPSs) and membrane lipids. Besides the barrier role of SPSs, additional mechanisms involving the activity of efflux pumps and the peptide uptake transporter BacA, together with the production of H_2_O_2_ and formaldehyde have been unveiled. Also, the induction of the iron-uptake machinery in both predator and prey reflects a strong competition for this metal. With this research we complete the characterization of the complex transcriptional changes that occur during the *M. xanthus*-*S. meliloti* interaction, which can impact the establishment of beneficial symbiosis with legumes.

## 1. Introduction

Bacteria interact with co-habiting microbes in different multispecies communities. The ecological and evolutionary success of microorganisms in a particular environment is not only governed by their capacity to adapt to external abiotic stresses, but also depends on their ability to detect and respond to competition with the neighboring cells. Consequently, the metabolic processes of one strain are influenced by the metabolic functions of the other members of the community. The relationships between microorganisms range from cooperative symbiotic associations to different competition strategies. In all cases, bacterial interactions involve complex processes that are key determinants that strongly shape the structure of bacterial communities (Bauer et al., 2018; Granato et al., 2019). In the last decades many studies have revealed how bacterial interactions that occur in small communities have consequences that affect in many cases human, animal, and plant health (Pérez et al., 2011; Stubbendieck et al., 2016; Niehaus et al., 2019; Molina-Santiago et al., 2021; Martins et al., 2022).

A particular type of interaction is represented by predatory bacteria, which are species that kill and lyse susceptible cells in order to consume the cellular materials as carbon and energy sources (Pérez et al., 2016; Whitworth et al., 2020). Most bacterial predators use two major approaches to kill prey: (i) the endobiotic strategy represented by *Bdellovibrio* and like organisms (BALOs) that mainly prey on diderm bacteria in the planktonic phase as well as in biofilms (Mookherjee and Jurkevitch, 2022), and (ii) the epibiotic predation exemplified by myxobacteria that can kill and externally lyse a great variety of microorganisms (Muñoz-Dorado et al., 2016; Pérez et al., 2016).

The capacity of bacterial predators to kill other bacteria, including multidrug-resistant pathogens, has attracted the attention of researchers as a feasible alternative to antibiotics in the actual crisis (Pérez et al., 2020). As the use of technologies such as next generation sequencing and meta-transcriptomics are increasingly being applied to the study of soil microbiota, bacterial predation is gaining relevant importance as a shaper of microbial communities. Although myxobacteria have been traditionally considered minority components of soil bacterial communities, several studies have revealed that this does not seem to be the case, and that the myxobacterial community is a predominant and highly diverse group within soil niches (Zhou et al., 2014). Until recently, protists have been considered the dominant group preying on bacteria. However, the results of recent studies strongly suggest the importance, and possibly even dominance, of myxobacteria as soil predators. In fact, an analysis of 28 European soils showed that in most of these soils myxobacteria comprise 1.5–9.7% of all obtained SSU rRNA transcripts and more than 60% of all identified potential bacterivores (Petters et al., 2021).

*Myxococcus xanthus* is a soil myxobacterium which has been extensively studied because of its unique complex lifecycle. This lifecycle consists of two stages: a vegetative growth stage in the presence of nutrients and/or prey (it is a facultative predator); and a developmental stage (with the formation of macroscopic fruiting bodies filled of myxospores) when nutrients are depleted (Muñoz-Dorado et al., 2016). It has a large genome which encodes all the genes that participate in the complex social and multicellular lifestyle exhibited during both growth and development (Goldman et al., 2006).

*Myxococcus xanthus* predation requires the participation of many weapons to kill and consume the prey, including a variety of hydrolytic enzymes, outer membrane vesicles, contact-dependent and independent elements, and the production of secondary metabolites such as antibiotics (Pérez et al., 2016; Thiery and Kaimer, 2020; Seef et al., 2021). In addition, this arsenal differs from one prey to another (Thiery et al., 2022).

In our laboratory we deciphered the transcriptomic changes that take place in *M. xanthus* during a complete lifecycle. During development, 1,415 genes were sequentially and differentially expressed in 10 discrete groups (Muñoz-Dorado et al., 2019). Moreover, we have also analyzed the predatosome of this myxobacterium when preying on *Sinorhizobium meliloti*. The results obtained revealed that the number of genes differentially expressed during predation is lower than during development. Among the transcripts that are up-regulated during predation, the most noteworthy are genes involved in the biosynthesis of secondary metabolites, in the synthesis and degradation of lipids, those encoding both extracellular and outer membrane hydrolytic enzymes, and genes related to social motility and Tad-like apparatuses (Pérez et al., 2022).

In predatory interactions, the prey will need to modify the network of genes and pathways required to mount an orchestrated defense against the biotic stress caused by the predatory attack. We will use the term “defensome” to refer to the whole set of genes that vary their transcription in response to the predatory bacterium, either to resist predation or to compete for resources. Nevertheless, some specific defense mechanisms and adaptations of different prey against *M. xanthus* attack have been reported, so far. For example, galactoglucan and melanin protect *S. meliloti* from predation by this myxobacterium (Pérez et al., 2014; Contreras-Moreno et al., 2020). The presence of this predator also induces the transcriptional activation of silenced genes coding for antibiotics in *Streptomyces coelicolor* (Pérez et al., 2011; Lee et al., 2020). *Bacillus subtilis* induces bacillaene synthesis and forms spore-filled megastructures against the attack of *M. xanthus* (Müller et al., 2014, 2015). On the other hand, studies of the prey response revealed a novel antibiotic resistance mechanism consisting of the glucosylation of the antibiotic myxovirescin TA, which was discovered in *Bacillus licheniformis* (Wang et al., 2019). Also, the transcriptome of *Escherichia coli* against *M. xanthus* in liquid media has been analyzed, and the results showed that the presence of the predator caused widespread induction in gene expression and enrichment of several pathways including ribosome production, lipopolysaccharide (LPS) biosynthesis, oxidative phosphorylation, production of antibiotics and secondary metabolites, energy and carbon metabolism, and vitamin and amino acid metabolism. However, only the pathway involved in glycerophospholipid metabolism was down-regulated (Livingstone et al., 2018).

In the current study, we have analyzed the defensome of the prey *S. meliloti* against attack by *M. xanthus*. *S. meliloti* is an alphaproteobacterium that establishes nitrogen-fixing symbiosis with legumes, thereby contributing to the fertility of soils. This soil bacterium can also exist as a free-living organism in natural environments, where it must adapt to diverse nutrient availability conditions, and compete with other neighboring microbes, including predators such as *M. xanthus*. These competitive interactions will affect not only the structure of the soils, but also, ultimately, will affect their fertility.

Our results reveal that the contact with *M. xanthus* induces in *S. meliloti* defense and/or adaptation genes that affect central pathways, such as protein biosynthesis and secretion. The reconstruction of other up-regulated pathways seems to indicate that *S. meliloti* not only protects itself against predator attack in a passive way by modifying the cell envelope, but also reacts actively by producing H_2_O_2_ or formaldehyde. The induction of genes related to iron uptake indicate ion competition. This research completes the study on transcriptomic changes undergone in both partners during the *M. xanthus*-*S. meliloti* interaction and draws a panoramic view of the mechanisms and pathways involved in attack, defense, and competition.

## 2. Materials and methods

### 2.1. Media, bacterial strains and growth conditions

*Sinorhizobium meliloti* Rm1021 (Meade and Signer, 1977) was used as prey, whereas *M. xanthus* DK1622 (Kaiser, 1979) was used as predator. Tryptone yeast (TY) solid and liquid media (Beringer, 1974) were used for maintenance and growth of *S. meliloti*. CTT solid and liquid media (Hodgkin and Kaiser, 1977) were used to grow *M. xanthus*, and for the predation experiments. Solid media contained 1.5% Bacto-Agar (Difco, Le Pont de Claix, France), and liquid cultures were incubated with vigorous shaking at 30°C.

### 2.2. Preparation of prey cells and co-culture of prey and predatory cells

*Sinorhizobium meliloti* was grown in TY broth to an optical density at 600 nm (OD_600_) of 1 and then diluted using the same broth to a final OD_600_ of 0.2. Twenty 10-μl drops of the diluted culture were deposited on the surface of CTT agar plates for each replicate and incubated at 30°C for 24 h. After that time, *S. meliloti* cells from two replicates were harvested from plates to obtain *t* = 0 prey samples (samples Sm_t0). Then, to obtain samples of predatory and prey interacting cells, 10-μl drops of *M. xanthus*, grown in CTT liquid media to an OD_600_ of 1 and concentrated in TM buffer [10 mM Tris-HCl (pH 7.6), 1 mM MgSO_4_] to a final OD_600_ of 15, were deposited on top of each of the rhizobial colonies of a subset of the plates (samples Mx_Sm). Another subset of samples of *S. meliloti* was kept growing alone (samples Sm). Two replicates from each of the two conditions (predator/prey co-culture and pure culture of *S. meliloti*) were harvested from plates after 2 and 6 h of incubation. Pellets from each sample were resuspended in 0.5 ml of RNA Protect Bacteria Reagent (Qiagen, Hilden, Germany), incubated at room temperature for 5 min, and centrifuged at 5,000 *g* for 10 min (4°C). Next, pellets were stored at −80°C.

### 2.3. RNA extraction

To purify RNA, cells were lysed for 10 min at room temperature with lysozyme and proteinase K [250 μl of 3 mg/ml lysozyme (Roche Diagnostic, Mannheim, Germany) and 0.4 mg/ml proteinase K (Ambion, Carlsbad, CA, United States)] prepared in TE buffer [10mMTris-HCl; 1mMethylenediaminetetraacetic acid (EDTA), pH 8.0]. RNeasy Mini Kit (Qiagen, Hilden, Germany) was used for RNA extraction [carrying out on-column DNase digestion with the RNAse-free DNase set (Qiagen, Hilden, Germany)], eluting each sample in 50 μl of RNase-free water.

### 2.4. Library preparation, sequencing, and global transcriptomic data analysis

Total RNA samples were processed by Novogene [Novogene Europe, Cambridge, United Kingdom], including rRNA depletion from total RNA samples with the Illumina Ribo-Zero Plus rRNA Reduction Kit (Illumina, Inc.). Remaining RNA was processed according to the procedures described in Pérez et al. (2022).

On average, 18.81 million raw reads and a coverage of 421.75x were obtained. After removing reads containing adapters, low quality reads and/or reads with more than 10 percent uncertain nucleotides, the genome coverage varied from 355.53x to 494.52x (median of 415.18x), which provide an excellent coverage of the mRNA fraction.

To facilitate comparison analyses with other *S. meliloti* transcriptomes we used the old nomenclature (SMa_, SMb_ or SMc_), since these identifiers are commonly used in the literature. However, the corresponding new localizers SM_RS are also indicated in the tables. To decipher the defensome, we have considered all the up- and down-regulated transcripts with | Log2 Fold Change| > 0 and *p*adj < 0.05. It must be taken into account that predation is a multifaceted process, and any change could be of interest as we have demonstrated in the case of *M. xanthus* predatosome (Pérez et al., 2022). The adaptation and defense mechanisms expected may not be very drastic and, for this reason, our research has been focused on those routes in which there is gene enrichment or in those pathways in which many of the genes involved are up or down regulated in the presence of the predator. In all figures and tables the Log2 Fold Change is specified and those Log2 Fold Change > 1 or Log2 Fold Change < −1 are highlighted. For researchers interested in this transcriptomics, all data including reads, fragments per kilobase of transcript per million fragments mapped (FPKMs) and Log2 Fold Change are attached as Supplementary material. However, for improved confidence, | Log2 Fold Change| > 1 has been used in comparisons with other transcriptomes, and this threshold is also indicated in all the figures and tables.

## 3. Results and discussion

### 3.1. Overview of the transcriptomic response of *S. meliloti* on predatory co-cultures

Transcriptional changes in *S. meliloti* in response to the predatory attack by *M. xanthus* was investigated by using the RNA-seq technology. The *M. xanthus*-*S. meliloti* co-culture conditions and the preparation of libraries have been previously described in detail (Pérez et al., 2022). We focused our study on two points in time after contact: at 2 and 6 h, a period of time during which the prey has to adapt its metabolism and structures to the biotic stress that represents the predatory attack. Following the same procedure used to elucidate the *M. xanthus* predatosome (Pérez et al., 2022), five cDNA libraries were constructed to analyze the *S. meliloti* defensome: Sm_t0: *S. meliloti* alone at time 0 h; Sm_t2: *S. meliloti* alone collected after 2 h on solid CTT medium; Sm_t6: *S. meliloti* alone grown for 6 h on solid CTT medium; Mx_Smt2: cells collected after 2 h of interaction between *M. xanthus* and *S. meliloti*; and Mx_Smt6: cells harvested after 6 h of the *M. xanthus*-*S. meliloti* interaction. From now on, the terms t2 and t6 will be used to refer to results obtained at 2 and 6 h of the co-cultures of *M. xanthus* and *S. meliloti* (Mx_Smt2 and Mx_Smt6, respectively) compared to their respective controls (prey cells in pure culture at 2 or 6 h: Sm_t2 and Sm_t6, respectively).

The total number of raw reads from each sample, the clean reads (obtained after removal of raw reads containing adapters and/or of low quality reads), the errors Q20 and Q30, and the GC content of the clean reads are compiled in Supplementary Table 1A.

The Pearson correlation coefficients (*R*2) between replicates were satisfactory in all samples (≥ 0.91) (Supplementary Figure 1A). The principal component analysis (PCA) showed that genes obtained from the same condition cluster together and genes from different nutritional stages and times cluster separately as expected (Supplementary Figure 1B). The RNA mean reads were normalized to FPKM values (Supplementary Table 1B). The FPKM density distributions and the violin diagrams showing similar gene expression levels are depicted in Supplementary Figures 1C, D, respectively.

**FIGURE 1:**
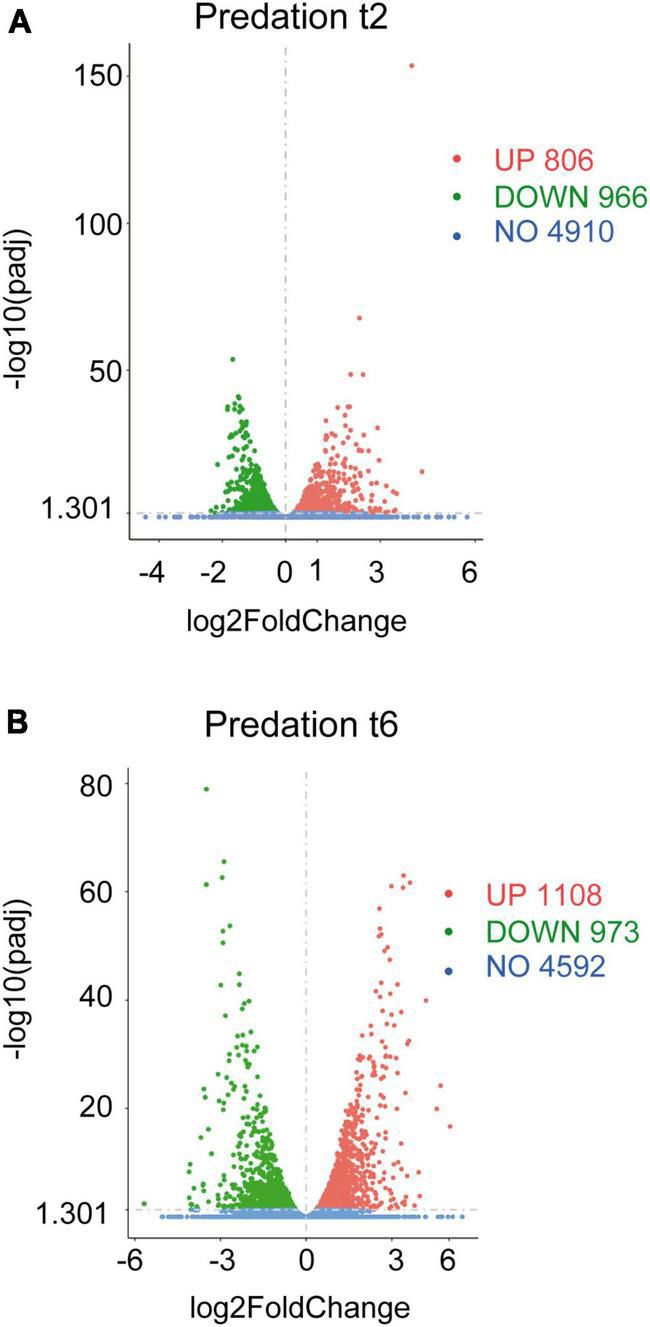
Differential gene expression of *Sinorhizobium meliloti* in response to *Myxococcus xanthus* predation. Volcano plots of up-regulated and down-regulated genes during the predatory interaction at **(A)** t2 and **(B)** t6 (2 and 6 h of contact). The estimated fold changes (*x*-axis) versus the minus log10 of the adjusted *p*-values (*y*-axis) from DESeq analysis are shown in the volcano plots. The significant genes with absolute values of | Log2 Fold Change| > 0 and *p*adj < 0.05 are depicted in red (up-regulated) or in green (down-regulated). Blue dots indicate non-regulated genes (NO). Gray vertical dotted lines indicate zero-fold change.

The volcano plots were constructed by using the transcripts of co-cultures at 2 and 6 h versus their respective controls filtered by their fold changes (| Log2 Fold Change| > 0) and *p*adj < 0.05 (Figures 1A, B).

Including novel genes and sRNAs (i.e., non-coding RNAs of 50–500 nt length), 1,772 and 2,081 transcripts were differentially regulated in *S. meliloti* in response to predator attack at 2 and 6 h, respectively (Figures 1A, B and Supplementary Tables 1C, D). Concerning the direction of transcript regulation, 806 transcripts (45.5%) were up-regulated and 966 (54.5%) down-regulated at 2 h, while 1,108 (53.2%), and 973 (46.8%) transcripts were up- and down-regulated, respectively, at 6 h. Overall, these data indicate that 26.5% (2 h) and 31.2% (6 h) of the *S. meliloti* transcriptome responded to predator attack. This represents a change in transcriptional activity notably greater than that observed in the predator under the same experimental conditions (Pérez et al., 2022). These results are in agreement with those obtained during predation on *E. coli*, where it was also found that predation caused a much more pronounced response in the prey than in the predator (Livingstone et al., 2018), indicating a strong adaptive, competitive and/or defensive response triggered by the presence of the predator. For further analyses of the defensome in this study, novel genes and sRNAs were not considered, leaving 1,361 and 1,818 transcripts as differentially regulated at 2 and 6 h, respectively. These genes have been organized in 973 up-regulated and 1,139 down-regulated genes identified at 2 h and/or 6 h in Supplementary Tables 2A, B.

We investigated whether predation alters gene expression in *S. meliloti* in a replicon-biased fashion. The *S. meliloti* Rm1021 genome (6.69 Mb in size) is composed of three large replicons: a chromosome (3.65 Mb) and two megaplasmids, pSymA (1.35 Mb), and pSymB (1.68 Mb) that contain 54, 21, and 25% of the total annotated genes (Galibert et al., 2001). We found that genes differentially regulated during predation were not proportionally distributed among the replicons. Instead, they were biased toward the chromosome (70.6%), whereas only 9.6 and 19.8% of the differentially expressed genes were associated to pSymA and pSymB, respectively (Supplementary Figure 2A). This chromosomal bias was mainly caused by genes whose expression was increased during predation since 83.2% were located on the chromosome with only 5.6% on pSymA and 11.2% on pSymB. In contrast, down-regulated genes were distributed more evenly, with 59.8% on the chromosome, 13% on pSymA and 27.2% on pSymB (Supplementary Figure 2B and Supplementary Table 3). These results indicate that in response to predator attack, *S. meliloti* activates chromosomal-encoded functions, whereas the symbiotic plasmids, especially pSymA, have a minor contribution.

To identify the main biological processes affected in *S. meliloti* during predation, two different approaches were used. In one of them, enrichment analyses were carried out using the associated pathways in the KEGG database (Kyoto Encyclopedia of Genes and Genomes; Kanehisa et al., 2021). The up-regulated pathways involved those related to ribosome production, oxidative phosphorylation, and biosynthesis of amino acids, secondary metabolites, and cofactors (Supplementary Figure 3A and Supplementary Tables 1E, F). The main down-regulated pathways during predation were related to valine, leucine, and isoleucine degradation, microbial metabolism in diverse environments, carbon metabolism, quorum sensing and ABC transporters (Supplementary Figure 3B and Supplementary Tables 1E, F). As a complementary approach, functional categories of the 973 up-regulated (Supplementary Table 2A) and the 1,139 down-regulated (Supplementary Table 2B) genes were determined using clusters of orthologous groups (COGs) according to the genome sequence annotations of the *S. meliloti* Genome Project^1^ (Table 1).

**TABLE 1:** Functional categories of genes differentially expressed in *Sinorhizobium meliloti* in response to predation by *Myxococcus xanthus*.

Gene category	Number of up-regulated genes^(1)^	Number of down-regulated genes
Not in COGs^(2)^	123 (43.5%)	160 (56.5%)
Function unknown	32 (28.3%)	81 (71.7%)
Translation	95 (88%)	13 (12%)
Intracellular trafficking and secretion	14 (87.5%)	2 (12.5%)
Cell wall/membrane biogenesis	52 (62.7%)	31 (37.3%)
Inorganic ion transport and metabolism	55 (61%)	35 (39%)
Nucleotide transport and metabolism	36 (61%)	23 (39%)
Secondary metabolites biosynthesis, transport, and catabolism	17 (50%)	17 (50%)
Transcription	49 (46.7%)	56 (53.3%)
Posttranslational modification, protein turnover, chaperones	37 (46.2%)	43 (53.8%)
Defense mechanisms	10 (43.5%)	13 (56.5%)
General function prediction only	76 (43.2%)	100 (56.8%)
Amino acid transport and metabolism	111 (42.7%)	149 (57.3%)
Cell motility	11 (42.3%)	15 (57.7%)
Replication, recombination, and repair	19 (42.2%)	26 (57.8%)
Energy production and conversion	63 (40.9%)	91 (59.1%)
Coenzyme transport and metabolism	26 (40%)	39 (60%)
Lipid transport and metabolism	33 (37.1%)	56 (62.9%)
Signal transduction mechanisms	16 (35.6%)	29 (64.4%)
Cell cycle control, mitosis and meiosis	7 (35%)	13 (65%)
Carbohydrate transport and metabolism	44 (23.2%)	146 (76.8%)

^(1)^Percent of up-regulated genes within each gene category is shown in parenthesis. Functional categories in which more than 60% of the genes differentially regulated show increased or reduced expression during predator attack are shown in light or dark gray, respectively.

^(2)^COG, cluster of orthologous groups.

Many differentially regulated genes are annotated as either exhibiting partial or global homology to genes deposited in the databases (Not in COGs) or having unknown functions. Nevertheless, several functional categories could be identified associated to up-regulated genes (highlighted in light gray in Table 1), or to down-regulated genes (highlighted in dark gray in Table 1). These analyses suggest that the *S. meliloti* response to the predatory attack by *M. xanthus* involves the activation of protein synthesis and secretion, increased energy generation, changes in the cell envelope and membranes, and the stimulation of the transport and metabolism of inorganic ions and nucleotides. At the same time, carbohydrate and lipid transport and metabolism, as well as signal transduction mechanisms and cell division-related functions are repressed during predation. Comparison of the *S. meliloti* defensome obtained in the present study with that of *E. coli* (Livingstone et al., 2018) reveals some similarities but also some differences. Thus, increased protein production and energy generation, and the biosynthesis of secondary metabolites were detected during predation on the two preys. However, whereas carbon metabolism and glycerophospholipid metabolism were up- and down-regulated, respectively, in *E. coli* during predation, the opposite regulation was found for the same functional categories in *S. meliloti*. If these differences reflect different evasion/defense strategies of the prey or if they are the result of different experimental approaches remains unknown.

### 3.2. Predation on *S. meliloti* activates protein production and secretion, fatty acid synthesis, and energy generation while repressing fatty acid degradation and carbohydrate transport and metabolism

A large fraction (88%) of the genes involved in translation which were identified as differentially regulated in the transcriptome profile exhibited increased expression differentially expressed genes involved in translation identified in our transcriptome exhibited increased expression during predation compared with control conditions (Table 1). Of the 95 up-regulated genes related to translation, 28 code for ribosomal proteins and proteins involved in ribosome maturation and modification, and 33 for aminoacyl-tRNA synthetases and proteins related with tRNA modification. In addition, genes putatively coding for translation initiation (*infA*, *infB*, *infC*) and elongation (*tsf*, *efp*) factors, as well as probable peptide chain release factors (*prfA*, *prfB*, *prfC*) were also up-regulated, strongly suggesting the activation of protein synthesis during predation. Hence, translation is a biological process activated during predation in different prey (Livingstone et al., 2018), but also in the predator (Pérez et al., 2022).

In agreement with increased protein synthesis and the consequent greater demand for amino acids, 111 genes involved in amino acid transport and metabolism were also up-regulated. Moreover, genes coding for the ATP-dependent chaperone folding system DnaK/DnaJ/GrpE exhibited increased expression. This system plays a crucial role in microbial proteostasis under both normal and stress conditions by assisting the folding of newly synthesized polypeptides, and of misfolded or aggregated proteins (Winter and Jakob, 2004; Barriot et al., 2020). Recently, *S. meliloti* DnaJ has been shown to participate in tolerance to different stresses (Brito-Santana et al., 2023). Besides assisting protein folding, the DnaK chaperone also facilitates protein targeting to membranes and protein translocation (Barriot et al., 2020). Interestingly, we found that the majority of genes involved in intracellular trafficking and secretion identified as differentially expressed during predation were also up-regulated (Table 1), suggesting increased protein secretion. This was the case for several genes related to the Sec system, which is responsible for the insertion, translocation, and secretion across the membrane of unfolded polypeptides, which carry a removable N-terminal signal sequence. Thus, genes coding for the membrane-embedded SecYEG translocon, the SecA ATPase and piloting factors such as the signal recognition particle (SRP) Ffh or the SecB chaperone, which maintain newly synthesized proteins in an unfolded conformation and drive them to the membrane (Papanikou et al., 2007), exhibited increased expression during predation. In contrast to the Sec translocon, the Tat system is responsible for exporting previously folded proteins which have a particular signal peptide containing a recognizable twin-arginine motif (Pickering and Oresnik, 2010; Palmer and Berks, 2012). These proteins seem also to be actively exported in *S. meliloti* during predation, as suggested by the up-regulation of the *tatA* gene coding for a TatA/E translocase homolog. The up-regulation of protein secretion systems has also been reported in myxobacteria, where it has been associated to the secretion of factors required for predation of bacteria and fungi (Li et al., 2019; Pérez et al., 2022). In the case of the prey, increased protein secretion could be required to maintain bacterial cell envelope protein complexes, whose integrity might be damaged during predatory attack.

**FIGURE 2:**
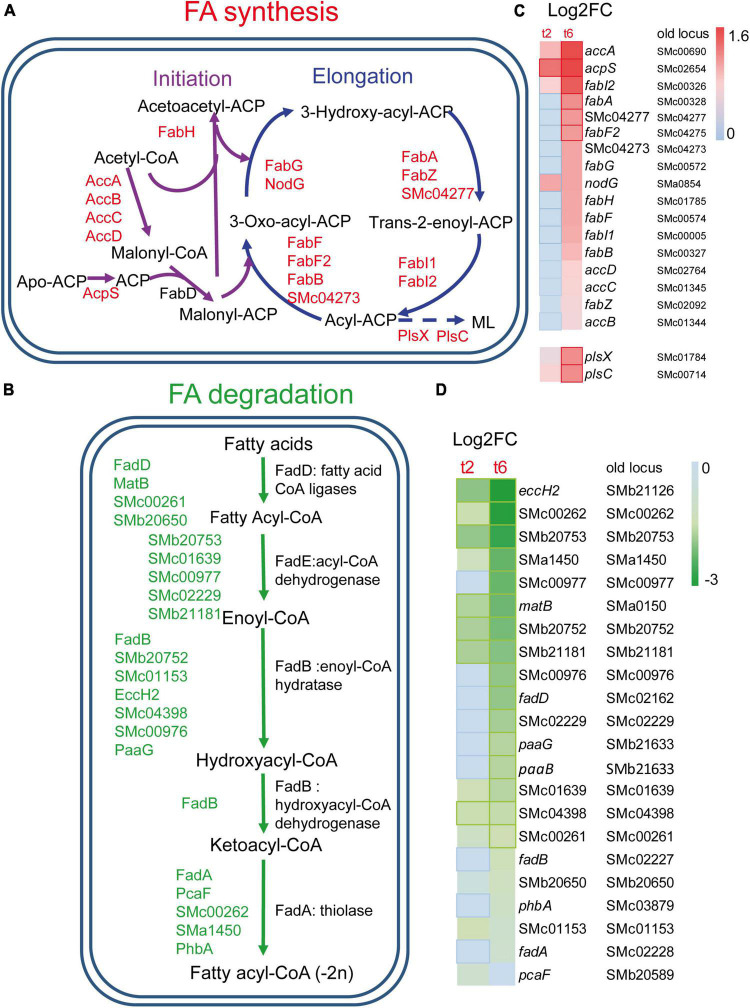
Changes in fatty acids (FA) metabolism in *S. meliloti* in co-cultures with *Myxococcus xanthus* after 2 and 6 h of contact (t2 and t6). **(A)** Up-regulation of genes involved in FA biosynthesis. ML, membrane lipids (see Figure 4 for more information). **(B)** Down-regulation of the genes responsible for the β-oxidation degradative pathway. Those genes (up or down-regulated) with demonstrated activity in the literature are indicated by their names (see text for details), while paralogous genes found in the KEGG database and that are also differentially expressed are represented by their gene identifiers. **(C,D)** Heatmaps of the genes involved in FA biosynthesis and FA β-oxidation, respectively. Red or green edges indicate genes with | Log2 Fold Change| > 1, and dotted edges indicate no differentially expressed genes at the indicated time.

Many genes coding for the type II fatty acid synthase (FAS II) system, which is responsible for FA synthesis, were found to be up-regulated in *S. meliloti* during predation by *M. xanthus* (Figures 2A, 2C). These include genes coding for most of the enzymes involved in the initiation phase and elongation cycles of FA chains (López-Lara and Soto, 2018), as well as some paralogous genes. Activation of FA synthesis is also suggested by the increased expression of *acpS* (*smc02654*), the holo-acyl-carrier protein (ACP) synthase, which transfers the 4’-phosphopantetheine from coenzyme A (CoA) to apo-ACP, thereby converting it to the functional holo-ACP to which acyl intermediates can be bound. The up-regulation of *fabA* and *fabB* required for unsaturated FA synthesis, together with that of *fabI* and *fabF*, suggests that the synthesis of both saturated and unsaturated FAs is increased during predation (López-Lara and Soto, 2018). In contrast, FA degradation decreased during predatory attack, as indicated by the down-regulation of several genes required for this process. Among them, genes coding for proteins involved in the uptake of long-chain FAs (*fadL*), genes involved in the activation of different FAs with CoA (*fadD*, *matB*, *smc00261*, *smb20650*), as well as genes of the *smc02229-fadA-fadB* operon, which most likely code for the enzymes required for the different steps in the β-oxidation cycle, are found (Figures 2B, 2D). Decreased FA degradation, together with increased FA biosynthesis, could indicate that *S. meliloti* requires FAs for a function other than energy production, perhaps for building and remodeling the cell membrane in response to predatory attack (see section “3.4. Drastic changes in the *S. meliloti* cell envelope during predation”). Lipid biosynthesis is also activated in *M. xanthus* during predation, most likely to change the lipid composition of the cell envelope and to synthesize new secondary metabolites that will contribute to kill prey. However, and in contrast to its prey, genes coding for enzymes involved in the β-oxidation cycle are up-regulated indicating an increased energy demand during predatory attack (Pérez et al., 2022).

Consistent with increased synthesis of proteins and FA, which are metabolically demanding processes, the up-regulation of up to 63 genes involved in energy production and conversion was detected. These include genes coding for different complexes of the respiratory chain and associated functions, such as the chromosomal *nuo* genes that code for the proton-pumping NADH: ubiquinone oxidoreductase (*nuoA1B1C1D1E1F1HIJK1LMN*), genes for cytochrome o ubiquinol oxidase (*cyoABC*), cytochrome c oxidase (*ctaCD*), cytochrome b (*fbcB*), and a putative ATP synthase (*atpBCF2*). Increased expression of genes that code for different enzymes of the tricarboxylic acid cycle was also detected (*pdhABC*, *lpdA1*, *sdhABCD*, *fumC*, *sucA*, *icd*, *acnA*, *mdh*).

Similar to genes involved in FA degradation, the majority of the genes belonging to the category of carbohydrate transport and metabolism (76.8%) were down-regulated in the transcriptome. Genes coding for different sugar ABC transporters (*ugpABC*, *frcAK*, *rhaST*), glucolytic enzymes (*pgm*, *pgi*, *pgiA1*, *cbbA*, *cbbA2*, *tpiA1*, *gap*, *pgk*, *pykA*), enzymes of the pentose phosphate pathway (*cbbT*, *gnd*, *eda2*, *rbsK*, *ttuD1*), and enzymes involved in glycogen (*glgC*, *glgA1*) and poly-3-hydroxybutyrate (*phbABC*) synthesis, showed reduced expression compared with control conditions. Disruption of the glycogen synthase gene *glgA1* resulted in decreased polyhydroxybutyrate (PHB) levels and increased EPS levels compared to the wild type (D’Alessio et al., 2017). In starving conditions, the use of glycogen could be an alternative energy source. In favor of this hypothesis is the fact that gene *glgX1*, involved in glycogen-debranching, is up-regulated.

### 3.3. Increased expression of genes related to iron and phosphorus starvation responses

**FIGURE 3:**
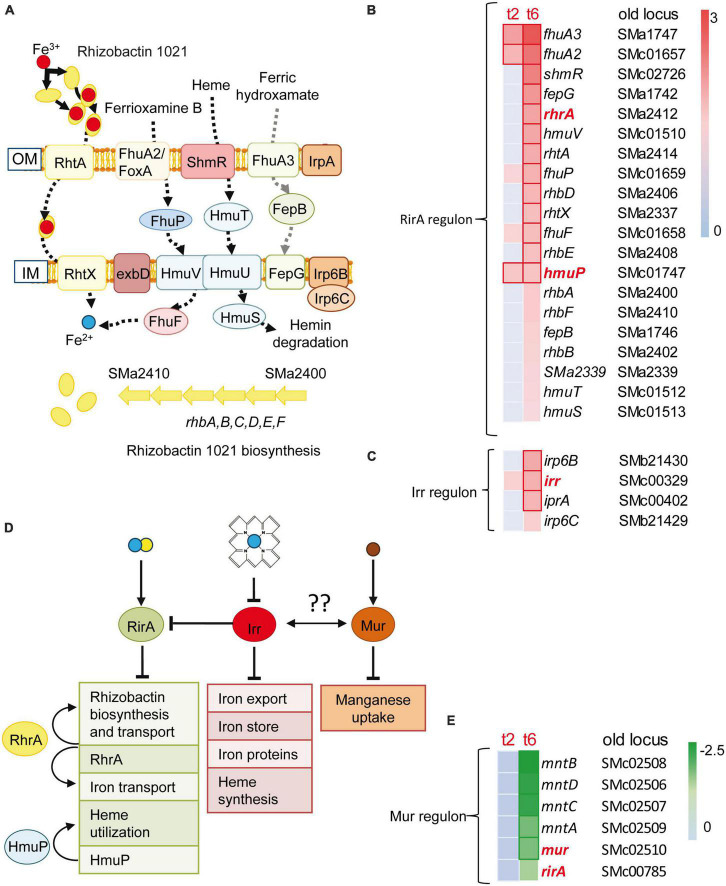
Iron uptake and rhizobactin 1021 biosynthesis are induced during competition. **(A)** *Sinorhizobium meliloti* genes involved in siderophore synthesis and iron-uptake regulation that are induced at t2 or/and t6. Red and blue circles represent Fe^3+^ and Fe^2+^, respectively. **(B,C)** Heatmaps of the RirA and Irr dependent genes which are depicted in panel **(A)**. **(D)** Control of iron homeostasis by the regulators: RirA, RhrA, HmuP and Irr. RirA is a (4Fe–4S) cluster containing protein which represses many genes involved in iron uptake under iron-replete conditions. The manganese responsive Fur-like repressor, Mur, controls manganese uptake. Both global regulatory proteins are down-regulated during predation, indicating a mechanism for the control of iron homeostasis by manganese as has been suggested for other bacteria (see text for details). (Fe–S) clusters are depicted as blue and yellow circles. Brown circles represent Mn^2+^. Arrows and truncated lines indicate positive and negative regulation, respectively. OM, outer membrane; IM, inner membrane. **(E)** Down-regulation of *rirA*, *mur* and Mur-dependent genes (see text, and Supplementary Tables 4A, B for details).

Inorganic transport and metabolism was a functional category that showed enrichment in up-regulated genes identified in the response to predatory attack (Table 1). Within this category, it was remarkable the up-regulation of many genes involved in iron uptake and metabolism, including genes involved in the synthesis (*rhbABDEF* and *sma2339*) and uptake (*rhtA*, *rhtX*) of the siderophore rhizobactin 1021 (Lynch et al., 2001). Additional genes related with iron and with increased expression are those involved in the uptake of heme and hydroxamate siderophores [*shmR*, *hmuSTV*, *fhuA2/foxA*, *fhuA3*, *fhuP*, *sma1742 fepG* (*sma1742*), *fepB* (*sma1746*)], the *exbD* gene encoding one of the components of the TonB-ExbB-ExbD complex, which provides the energy for the transport of heme and siderophore-mediated iron transport across the outer membrane, the *fhuF* gene coding for ferrioxamine B reductase, or genes for putative Fe^3+^ ABC transporters [*irp6C* (*smb21429*) and *irp6B* (*smb21430*)] (Fabiano and O’Brian, 2012; O’Brian, 2015) (Figures 3A–C). Genes coding for regulatory proteins involved in iron homeostasis (Supplementary Figures 4A, B and Supplementary Tables 4A, B) were also differentially expressed during predatory attack. Genes coding for HmuP, which controls hemin acquisition, RhrA, which controls both the synthesis and the uptake of rhizobactin 1021, and the iron regulator Irr were up-regulated, whereas the gene coding for the rhizobial iron regulator A (RirA) was down-regulated (Amarelle et al., 2010; O’Brian, 2015). Irr senses iron indirectly through the status of heme biosynthesis (Figure 3D). Under iron-limiting conditions, Irr represses genes encoding proteins that function under iron-sufficient conditions, including *rirA* (O’Brian, 2015). RirA is an iron-sulfur protein that acts as a repressor of iron-uptake under iron-replete conditions (Chao et al., 2005; Pellicer Martinez et al., 2017; Figure 3B). Therefore, data obtained here indicate that *S. meliloti* is experiencing iron-limiting conditions during co-culture with the myxobacterial predator, similar to the situation previously reported for *S. coelicolor* (Lee et al., 2020) and *Pseudomonas putida* (Akbar and Stevens, 2021). Up-regulation of the iron-uptake machinery was also observed in *M. xanthus* during co-culture with *S. meliloti* (Pérez et al., 2022), suggesting that both predator and prey are competing for iron. It would be interesting to test whether increased rhizobactin 1021 production in *S. meliloti* Rm1021 enhances rhizobial resistance to myxobacterial predation as shown for *P. putida* survivors that overproduce the siderophore pyoverdine (Akbar and Stevens, 2021).

It is known that the metabolism of iron and manganese are interrelated (O’Brian, 2015). Manganese can replace iron in many enzymes. However, and despite experiencing low iron concentrations, the expression of the *mntABCD* (formerly *sitABCD*) genes coding for a manganese ABC transporter (Platero et al., 2003) and that of its regulator Mur were down-regulated during predation (Figure 3E).

Several genes related with phosphorous uptake and metabolism were up-regulated in the defensome of *S. meliloti*, such as those coding for phosphate ABC-type transporters (*phoCDE*, *pstA*), phosphonate metabolism (*phnGHIJ*), alkaline phosphatase (*phoX*), polyphosphate kinase (*ppk*), as well as genes coding for regulatory proteins that are crucial for the maintenance of cellular phosphate homeostasis (*phoB* and *phoU*). Intriguingly, up to 29 of the up-regulated genes in the defensome were also found to be up-regulated in the phosphate starvation response of *S. meliloti*, with the majority of them (25) belonging to the cluster I of PhoB-dependent genes induced by phosphate stress (Supplementary Figure 4B and Supplementary Tables 4C, D; Krol and Becker, 2004). PhoB is the response regulator of the PhoR-PhoB two-component system that controls a set of genes known as the Pho regulon, which is involved in cell adaptation to phosphate starvation. In addition to genes related to phosphorus uptake and metabolism, the Pho regulon includes other genes such as those related to phosphorus-free membrane lipid biosynthesis (*sqdB*, *btaAB*, *olsA*, *olsB*), which were also up-regulated during predatory attack. Moreover, 29 genes up-regulated in the defensome increased their expression levels under phosphate starvation in a PhoB-independent manner. This group includes several genes involved in the synthesis of the exopolysaccharide I (EPS I) or succinoglycan (*exoA*, *exoW*, *exoV*, *exoK*, *exoYF1*, *exoX*) (see also Section “3.4. Drastic changes in the *S. meliloti* cell envelope during predation”). The transcriptional activation of low phosphate-responsive genes, including members of the Pho regulon, could be interpreted as *S. meliloti* cells facing phosphate-limiting conditions. However, this is difficult to believe considering that the medium used in the experimental setup is a phosphate-rich medium and that *phoU* is transcriptionally up-regulated. It has been suggested that the regulatory protein PhoU responds to elevated phosphate levels by significantly decreasing the phosphate transport of PstSCAB to prevent phosphate toxicity (diCenzo et al., 2017). Moreover, the activation of EPS I production is known to occur under high-phosphate conditions (Mendrygal and González, 2000; Acosta-Jurado et al., 2021). Therefore, and most likely, the up-regulation of low phosphate-responsive genes in the defensome cannot be the direct result of a phosphorous deficiency. In *E. coli*, PhoB is not only activated by low phosphate, but also by cell envelope stress (Choudhary et al., 2020). It is not unreasonable to think that compounds and hydrolytic enzymes secreted by *M. xanthus* during predatory attack disrupt different components of the *S. meliloti* cell envelope, causing cell envelope stress. Activation of the PhoB regulator by the cell envelope stress caused during predatory attack and/or any other mechanism, together with the known overlap and interaction of the Pho regulon with several other control circuits (Yuan et al., 2006), could explain the differential expression of low phosphate-induced genes during myxobacterial predation.

### 3.4. Drastic changes in the *S. meliloti* cell envelope during predation

The bacterial cell envelope is a complex structure that provides structural integrity and protects the cytoplasm from changes in the surrounding environment. As a diderm bacterium, the rhizobial cell envelope consists of three layers: the cytoplasmic or inner membrane (IM), a thin peptidoglycan cell wall, and the outer membrane (OM) containing LPS in the outer leaflet. In addition, rhizobia produce different surface polysaccharides (SPSs) that serve different functions during the free-living and symbiotic lifestyles of these bacteria (Acosta-Jurado et al., 2021). Maintenance of cell envelope integrity is essential for viability, and bacteria have developed regulatory mechanisms to defend from envelope disturbance. Considering the epibiotic predatory strategy employed by *M. xanthus*, it is not surprising that many *S. meliloti* genes related with the different cell envelope structures are transcriptionally modulated in response to predator attack. Below, we provide interpretation for the transcriptional changes detected in genes associated to different cell envelope structures.

In *S. meliloti*, the *exo/exs* and *wgx* (formerly *exp*) genes are responsible for the production of two different kinds of acidic exopolysaccharides (EPSs): EPS I or succinoglycan, and EPS II or galactoglucan (Becker et al., 2002). The regulation of these EPSs is very complex, and several environmental conditions and regulatory proteins have been involved in their control (Barnett and Long, 2018). As previously mentioned, several *exo/exs* genes involved in the synthesis of EPS I (*exoA*, *exoB*, *exoQ*, *exoF1*, *exoH*, *exoK*, *exoX*, *exoV*, *exoY*, *exoN*, *exoP*, *exoW*, *exoK*, *exsA*, *exsI*) (Glucksmann et al., 1993) were found to be up-regulated during predation. Concerning EPS II, the *S. meliloti* strain Rm1021 used in this study lacks a functional ExpR, a LuxR-type regulator that is required for the quorum sensing-dependent production of high amounts of EPS II (Pellock et al., 2002). Nevertheless, the increased expression of *wgcA* (*expC*), *wgdA* (*expE1*) and that of the *wggR* (*expG*) gene that codes for the transcriptional activator of EPS II-related genes, suggest stimulation of EPS II synthesis. Transcriptional activation of EPS II-related genes could be mediated by PhoB, which activates expression of *wggR* (Bahlawane et al., 2008). As discussed in section “3.3. Increased expression of genes related to iron and phosphorus starvation responses,” the up-regulation of EPS I-related genes is independent of PhoB. In this case, the transcriptional activation of the *exo/exs* genes could be mediated by regulatory proteins and circuits known to influence EPS I production and whose expression was also induced during predation. This is the case of *mucR* (Bahlawane et al., 2008), the *exoS*/*chvG* gene of the ExoR-ExoS-ChvI system (Yao et al., 2004; Wells et al., 2007). and the *ntrY* and *ntrX* genes of the NtrY-NtrX regulatory system (Calatrava-Morales et al., 2017). Recently, the activation of the ChvG/ExoS-ChvI regulon in response to cell wall stress has been suggested for Alphaproteobacteria (Williams et al., 2022), as well as the co-ordinated work of the ExoR-ExoS-ChvI and NtrY-NtrX systems to control different functions, including regulation of the bacterial cell envelope (Stein et al., 2021).

In addition to EPS I and EPS II, the production of other SPSs seems to be activated in *S. meliloti* during predation. This is the case for the low-molecular mass K polysaccharide (KPS) (Fraysse et al., 2005), and an as-yet-uncharacterized SPS that confers resistance to the antibiotic phazolicin (PHZ) and provisionally named PPP (PHZ-protecting polysaccharide) (Travin et al., 2023). Several genes related with the production of KPS (*rkpG*, *rkpI*, *rkpZ2*, *kpsF3*, *rkpU*, *rkpT2*, *rkpS*) and seven out of the nine genes located between *smb21252* and *smb21271* that are necessary for the synthesis of PPP are up-regulated during predatory attack, suggesting that these two SPSs could also contribute to the defense against predation. KPS biosynthetic genes were also found to contribute to PHZ resistance in *S. meliloti*, although to a lesser extent than genes involved in PPP synthesis (Travin et al., 2023).

In rhizobia, SPSs contribute to protect cells against abiotic factors and defense products produced by the plant and other microbes (Soto et al., 2006; Lehman and Long, 2013; Arnold et al., 2017; Travin et al., 2023). Production of increased levels of different SPSs during predation might be a defense strategy of the prey, in which SPSs play a barrier role by sequestering and/or repulsing hydrolytic enzymes and toxic compounds released by the predator. Indeed, earlier findings showed that the production of high levels of EPS II, characteristic of *S. meliloti* ExpR-plus strains, contributes to rhizobial resistance to myxobacterial predation (Pérez et al., 2014). In support of an important role of SPSs for defense against predators, a recent study associated increased alginate production and mucoidy as a strategy in *P. putida* to survive predation by another myxobacterial predator, *Cystobacter ferrugineus* (Akbar and Stevens, 2021).

**FIGURE 4:**
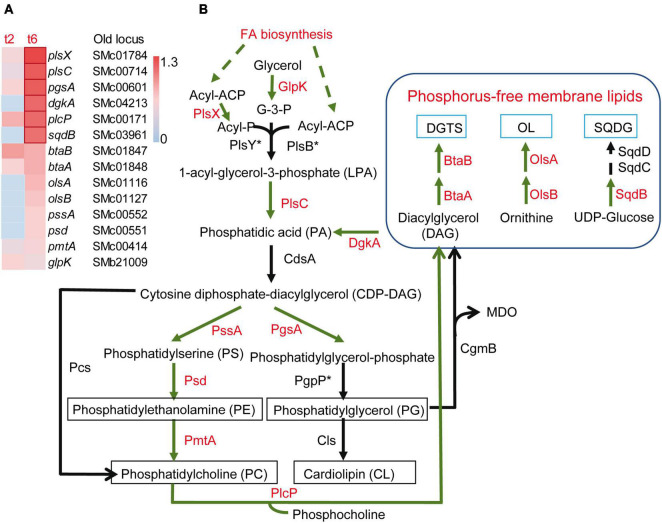
Increased expression of genes involved in membrane lipid formation in *Sinorhizobium meliloti* in co-culture with *Myxococcus xanthus* after 2 and/or 6 h of contact (t2 and/or t6). **(A)** Heatmap of up-regulated genes encoding different enzymes involved in membrane lipid formation. Red edges indicate genes with Log2 Fold Change > 1. **(B)** Metabolic routes for the synthesis of phospholipids and phosphorus-free membrane lipids. Up-regulated processes (FA biosynthesis) and enzymes exhibiting increased expression in *S. meliloti* during predation are shown in red. The asterisk indicates that the corresponding orthologous gene in *S. meliloti* has not been identified. DGTS, diacylglyceryl-N,N,N-trimethylhomoserine; OL, ornithine-containing lipids; SQDG, sulphoquinovosyl diacyl-glycerol; GlpK, glycerol kinase; PlsX/PlsY/PlsC and PlsB/PlsC are two different acyltransferase systems for the formation of PA, with the former most likely operating in *S. meliloti*; CdsA, CDP-DAG synthase; PssA, PS synthase; Psd, PS decarboxylase; PmtA, PE methyltransferase; Pcs, PC synthase; PgsA, PG-phosphate synthase; PgpP, PG-phosphate phosphatase; Cls, cardiolipin synthase; PlcP, phospholipase C; CgmB, cyclic glucan-modifying phosphoglycerol transferase; BtaA, S-adenosylmethionine: DAG 3-amino-3-carboxypropyl transferase; BtaB, diacylglyceryl homoserine N-methyltransferase; OlsA, *O*-acyltransferase; OlsB, lyso-ornithine lipid synthase; SqdB, UDP-sulfoquinovose synthase; SqdC, epimerase; SqdD, glycosyltransferase. Asterisks indicate that the corresponding genes have not been annotated in the *S. meliloti* Rm1021 genome.

Besides the activation of SPS-related genes, many loci coding for enzymes that affect the peptidoglycan and structural components of the OM and IM were differentially expressed during co-culture with the myxobacterial predator, suggesting modifications of the cell envelope. Some genes related with peptidoglycan synthesis and remodeling were up-regulated, such as *mltB2*, *murA*, *murC*, *murE*, whereas *murD*, *murF*, *murG*, or *pbpC*, were down-regulated. The expression of a set of *S. meliloti* genes related with the synthesis of the lipid A, the core polysaccharide and the O-antigen of LPS (*acpXL*, *smc04277-fabF2-smc04273*, *lpxXL*, *lpxK*, *ddhAB*, *kdsB*, *kdtA*, *lpsB*, *lpsCE*, *lpsS*) (Lagares et al., 2001; Sharypova et al., 2003), as well as the *lsrB* gene that codes for a transcriptional regulator that activates the expression of LPS biosynthetic genes (Tang et al., 2014), were found to be up-regulated during predation. Likewise, the periplasmic TolB1 and the OM-anchored Pal lipoprotein, which are elements of the Tol/Pal system known to contribute to cell envelope integrity (Krol et al., 2020; Szczepaniak et al., 2020), showed increased expression. It was also remarkable that several genes involved in membrane lipid synthesis were up-regulated (Figures 4A, B). Under phosphate-sufficient conditions, *S. meliloti* produces the glycerophospholipids phosphatidylglycerol (PG), phosphatidylethanolamine (PE), phosphatidylcholine (PC) and cardiolipin (CL) as its major membrane lipids (Sohlenkamp and Geiger, 2016; López-Lara and Geiger, 2017). However, when phosphate levels are limiting, *S. meliloti* replaces its phospholipids with membrane lipids that do not contain any phosphorus, namely sulphoquinovosyl diacyl-glycerols (SQDG), diacylglyceryl-N,N,N-trimethylhomoserines (DGTS), and ornithine-containing lipids (OL) (López-Lara and Geiger, 2017). Intriguingly, in response to myxobacterial predation, *S. meliloti* increased the expression of genes required for the synthesis of both phospholipids (*glpK*, *plsX*, *plsC*, *pgsA*, *pssA*, *psd*, *pmtA*) and phosphorous-free membrane lipids (*sqdB*, *btaAB*, *olsB*, *olsA*) (Figures 4A, B). This contrasts with the down-regulation of glycerophospholipid metabolism observed in the defensome of *E. coli* against *M. xanthus* in liquid media (Livingstone et al., 2018). In the defensome of *S. meliloti*, the up-regulation of the *plcP* gene was also detected. This gene codes for a phospholipase C that degrades PC to release diacylglycerol (DAG) that can be used for DGTS synthesis (Zavaleta-Pastor et al., 2010). As with the genes coding for the enzymes participating in the synthesis of phosphorous-free membrane lipids (see section “3.3. Increased expression of genes related to iron and phosphorus starvation responses”), *plcP* is also regulated by PhoB under phosphorus limitation (Krol and Becker, 2004; Yuan et al., 2006; Zavaleta-Pastor et al., 2010). However, under our experimental conditions and as discussed in the previous section, it is very unlikely that low levels of phosphate are responsible for the induction of these genes during myxobacterial predation. Bacteria change their membrane lipid composition in response to environmental changes and abiotic stresses (Sohlenkamp and Geiger, 2016). Our transcriptomic data suggest that during predation, *S. meliloti* undergoes membrane remodeling in which the formation of several membrane lipids, including those devoid of phosphorous, is activated under phosphorous-sufficient conditions. If this is simply a consequence of predation stress or represents an adaptation strategy that provides a fitness advantage by allowing the prey to conserve phosphate for other cellular processes and/or by stabilizing their membranes, requires experimental investigation.

### 3.5. Predatory stress and activation of defense mechanisms

Some features already described in previous sections, such as the unexpected activation of the PhoB regulon under phosphorous-sufficient conditions, the activation of the ExoS-ChvI and NtrY-NtrX regulatory systems, and the dramatic changes that the *S. meliloti* cell envelope is experiencing during co-culture with *M. xanthus*, are indicative of predatory stress responses. The up-regulation of *rsiB1* and *relA* also detected in the transcriptome are compatible with this notion. RsiB1 is one of the components of the partner switching mechanism that controls the activity of the general stress response (GSR) σ factor RpoE2. RsiB1 is the anti-anti-σ factor, which binds the anti-σ RsiA1, thereby releasing the σ factor RpoE2, which alters the transcription of a large set of genes in response to several stresses (Bastiat et al., 2010). In *S. meliloti*, another σ factor, RpoH1, that regulates the expression of genes encoding the cell wall biosynthetic machinery and membrane biogenesis proteins (de Lucena et al., 2010), has been implicated in the regulation of membrane stress (Nicoud et al., 2021). Intriguingly, *rpoH1* was found to be down-regulated in the defensome. Therefore, the contribution of RpoE2 and RpoH1 in the defense of *S. meliloti* requires further investigation. In addition, the up-regulation of *relA* suggests the activation of the stringent response, a complex bacterial regulatory mechanism that aids the adaptation to stressful conditions (Irving et al., 2021). In *S. meliloti*, RelA is involved in the synthesis and hydrolysis of the bacterial second messenger (p)ppGpp that plays a crucial role in the stringent response. *S. meliloti relA* mutants have pleiotropic phenotypes, including the overproduction of EPS I (Krol and Becker, 2011; Wippel and Long, 2019).

**FIGURE 5:**
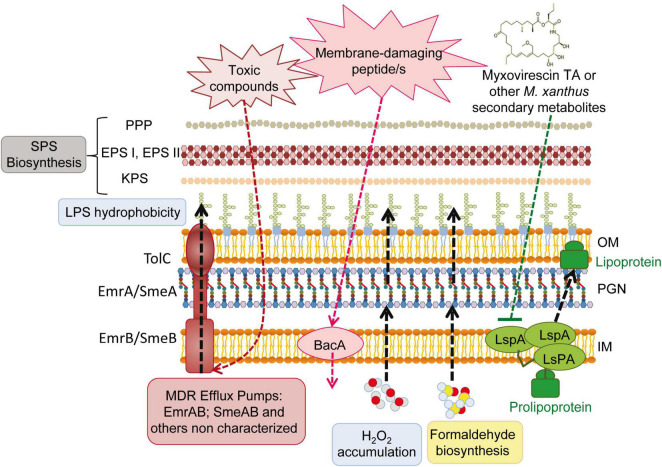
Overview of the deduced *Sinorhizobium meliloti* active and passive responses during the interaction with the predator *Myxococcus xanthus*. SPS, surface polysaccharides; PPP, PHZ-protecting polysaccharide; EPS, exopolysaccharides; EPS I, succinoglycan; EPS II, galactoglucan; KPS, K polysaccharide; LPS, lipopolysaccharide; OM, outer membrane; IM, inner membrane; PGN, peptidoglycan.

Together with the passive protective role of increased SPS production (see section “3.4. Drastic changes in the *S. meliloti* cell envelope during predation”), the overexpression of several efflux and transport proteins detected in this transcriptome could contribute to defend *S. meliloti* cells from predatory attack (Figure 5). Genes coding for the multidrug efflux systems EmrAB (Santos et al., 2014) and SmeABR (Eda et al., 2011), as well as the gene coding for the putative multidrug resistance EmrE protein, were found to be up-regulated during the co-culture. Multidrug resistance efflux pumps (MDR) enable bacteria to avoid the effects of a wide array of toxic compounds. The expression of the *emrAB* genes, which code for a putative major facilitator superfamily-type efflux pump, has been shown to increase in response to different abiotic stresses (e.g., heat shock, acidic pH) and a role for this efflux pump in the response to stresses by acting on the cell envelope has been suggested (Santos et al., 2014). In the case of SmeAB, this pump has been shown to confer resistance to different toxic compounds, including antibiotics, dyes, detergents, and antimicrobials produced by the host plant (Eda et al., 2011). Increased expression of the TetR-like regulator SmeR, which negatively regulates expression of SmeAB (Eda et al., 2011), suggests that the effector that releases the repression of SmeAB mediated by SmeR is being produced in the co-culture of *S. meliloti* with *M. xanthus*. Like *S. meliloti*, *P. putida* survivors to a different myxobacteria predation also exhibited increased expression of several efflux pumps (Akbar and Stevens, 2021), suggesting that this could be a common response used by prey to defend against predatory attack.

One of the transporters whose expression was up-regulated during *M. xanthus* predation was the broad-specificity peptide uptake transporter BacA (Figure 5). BacA is a SLiPT (SbmA-like peptide transporter) (Ghilarov et al., 2021), which was identified thirty years ago as an essential determinant in *S. meliloti* to establish a chronic infection inside the nodules of its host plant (Glazebrook et al., 1993). Years of investigation revealed that BacA is responsible for the uptake of nodule-specific cysteine-rich peptides (NCRs), defensin-like antimicrobial peptides that are produced inside the legume nodules and drive terminal bacteroid differentiation (Nicoud et al., 2021). With the internalization of NCRs, BacA protects rhizobial cells from the membrane-damaging activity of the host-produced peptides (Haag et al., 2011). Activation of *bacA* expression in response to predation could be indicative of the presence of as yet unknown membrane-damaging peptides released directly or indirectly by the predator.

The resistance to NCRs and other antimicrobial peptides is multifactorial (Arnold et al., 2017; Nicoud et al., 2021). In addition to BacA and the production of SPSs, modifications of the *S. meliloti* LPS mediated by LpsB and LpxXL have also been shown to contribute to resistance to NCR peptides (Nicoud et al., 2021). These genes code for a glycosyltransferase participating in the synthesis of the LPS core and a very-long-chain fatty acid (VLCFA; C ≥ 28) acyltransferase involved in the biosynthesis of lipid A, respectively (Figure 5). The unusual LpxXL-dependent acylation of the LPS is expected to increase the thickness of the OM and to increase lipid A hydrophobicity, which in turn reduces membrane fluidity. By increasing lipid A hydrophobicity, bacteria hamper the damaging effects of antimicrobial peptides on the membrane and contribute to increase resistance (Dalebroux and Miller, 2014). Interestingly, and as already mentioned in section “3.4. Drastic changes in the *S. meliloti* cell envelope during predation,” up-regulation of the *lpsB* and *lpxXL* genes was detected in response to predation. This, together with additional common transcriptional responses detected in the defensome and in the *S. meliloti* response to sublethal levels of NCR peptides (Penterman et al., 2014; see also next section “3.6. Transcriptional regulatory proteins that are differentially regulated”), supports the hypothesis of a rhizobial defense against putative predator-derived antimicrobial peptides.

Increased expression of the *smc01129* gene, encoding a probable lipoprotein signal peptidase, LspA, could be another mechanism of predation resistance, more specifically against the antibiotic myxovirescin TA produced by *M. xanthus*, which targets bacterial type II signal peptidase to inhibit pro-lipoprotein processing (Figure 5; Wang et al., 2019). The overexpression of the *lspA* gene has been shown to confer TA resistance to *E. coli* cells (Xiao et al., 2011). Although the myxovirescin genes are not up-regulated in the *M. xanthus* predatosome against *S. meliloti*, the role of this antibiotic in the interaction should not be excluded, since its overproduction could be regulated by a post-transcriptional mechanism (Pérez et al., 2022).

Formaldehyde secretion has been described as a potential predator-resistance feature of *Pseudomonas* species (Sutton et al., 2019; Akbar and Stevens, 2021). Interestingly, in the defensome of *S. meliloti* during myxobacterial predation, the up-regulation of different subunits of sarcosine oxidase (*soxA2*, *soxB2*, and *soxD2*), together with the down-regulation of the *gfa* (*smb20186*) gene, were observed. Considering that sarcosine oxidase catalyzes the oxidative demethylation of sarcosine to yield formaldehyde, and that Gfa is a putative glutathione-dependent formaldehyde-activating enzyme that participates in the formaldehyde detoxification pathway, the observed transcriptional changes could lead to the increased production and accumulation of formaldehyde in *S. meliloti* cells. Sarcosine oxidase activity also leads to glycine and H_2_O_2_ production. This, together with the observed up-regulation of the superoxide dismutase-encoding gene *sodB* that generates H_2_O_2_, and the down-regulation of the *katB* gene that codes for a catalase that inactivates H_2_O_2_, suggests a possible accumulation of this reactive oxygen species as an additional defense mechanism used by *S. meliloti* to fight against myxobacterial predators (Figure 5).

### 3.6. Transcriptional regulatory proteins that are differentially regulated

Regulatory proteins whose expression levels change in response to the predator should, directly or indirectly, be involved in controlling the expression of those genes and pathways involved in adaptation, competition, or defense that have been discussed above. We have found 49 regulators that increase their expression during predation, out of which, 11 are two-component systems (TCSs), and the rest are one-component regulators (including 5 GntR, 3 TetR, 3 MarR, and 3 LysR) (Supplementary Table 5A). In contrast, 76 regulatory elements have been found that are down-regulated (including 9 FCD, 6 LacI, 4 LysR, and 17 forming part of TCSs) (Supplementary Table 5B). Eight of them are related to symbiosis and/or induced in bacteroids. As discussed in section “3.3. Increased expression of genes related to iron and phosphorus starvation responses” (Figure 3), the Fur type manganese-responsive regulator, Mur, and the transcriptional regulator of the iron limitation response, RirA, are down regulated. Finally, SoxR, could be regulating a global response against oxidative-generating agents and might provide broad antibiotic resistance as has been described in *E. coli* (Hidalgo and Demple, 1997).

Elucidation of the role of these regulatory elements during predation will help not only to draw a global picture of the response of *S. meliloti* to predation, but also will shed light on adaptation processes caused by bacterial interactions.

### 3.7. Comparison of the *S. meliloti* defensome with different transcriptome profiles obtained under stress conditions

As already mentioned above, significant transcriptional changes occurring during co-culture with *M. xanthus* indicate that *S. meliloti* is experiencing stress when interacting with the predator. To identify putative common and/or differential mechanisms used by *S. meliloti* to cope with predatory stress and unfavorable conditions, we decided to compare the profile of the defensome with the transcriptome profile from cells grown under different abiotic stresses (osmotic, acid, or oxidative stress) (Domínguez-Ferreras et al., 2006; Hellweg et al., 2009; Lehman and Long, 2018), or treated with sublethal doses of NCR peptides (Penterman et al., 2014). To avoid noise, for all these comparisons, only differentially expressed transcripts in the defensome showing a | Log2 Fold Change| > 1 were considered.

Significant overlap was found between differentially expressed genes in the defensome and in response to abiotic stresses, especially under osmotic and oxidative stress. Intriguingly, the majority of the overlapping genes exhibited opposite regulation, suggesting different physiological adaptations of cells to abiotic or predatory stress (Supplementary Figures 5A–C and Supplementary Tables 6A–F). Among these genes, it was remarkable the differential regulation of many housekeeping genes involved in translation, and energy production and conversion, which were down-regulated under osmotic and oxidative stress conditions, but up-regulated during predation. Thus, whereas adaptation to abiotic stress involves a general slowing down of metabolism, the bacterial response to predatory attack requires activation of anabolism, especially protein and fatty acid synthesis.

A large overlap was also found between the defensome and genes differentially expressed in NCR-treated cells. Indeed, out of the 902 genes significantly altered in *S. meliloti* by NCR peptide treatment (Penterman et al., 2014), 180 genes were also found to be differentially expressed in response to myxobacterial predation (Supplementary Figure 5D and Supplementary Tables 6G, H). Approximately, 50% of the overlapping genes (91 genes) exhibited opposite regulation in both transcriptomes, but in this case, this group comprises genes down-regulated in the defensome and up-regulated in NCR-treated cells. Genes in this list include many genes involved in amino acid and carbohydrate transport and metabolism, energy production and conversion, and genes encoding proteins of unknown function. Interestingly, the catalase-encoding gene *katB* was included in this group, suggesting a putative H_2_O_2_ accumulation as a specific response to myxobacterial predation. Intriguingly, and contrasting with the results obtained comparing the defensome with the transcriptome profile of cells grown under abiotic stress, a greater number of genes (42) were found to be up-regulated in response to both NCR treatment and myxobacterial predation. This group comprises several genes involved in EPS synthesis and inorganic ion uptake (iron and potassium). Exposure of *S. meliloti* cells to cationic peptides leads to strong induction of the ExoS/ChvI, FeuP/FeuQ, and RirA regulons (Penterman et al., 2014). The transcriptomic data indicate that predatory attack activates ExoS/ChvI- and RirA-regulated genes (see sections “3.3. Increased expression of genes related to iron and phosphorus starvation responses” and “3.4. Drastic changes in the *S. meliloti* cell envelope during predation”). However, no significant overlap was found with genes regulated by the FeuP/FeuQ system that controls cyclic glucan production (Griffitts et al., 2008), suggesting that the Feu regulon is not relevant in the *S. meliloti* response to predatory attack, at least during the early stages of the interaction.

In an earlier transcriptome profile of *S. meliloti* cells exposed to 2 cationic NCR peptides, the down-regulation of genes involved in basic cellular functions, including translation, energy production, and fatty acid synthesis, was detected few minutes after treatment (Tiricz et al., 2013). This contrasts with the up-regulation of the same functional categories in response to predation. Nevertheless, some overlapping genes have been found between the study by Tiricz et al. (2013) and the defensome, including genes involved in stress responses (*dnaJ*, *ipbA*), iron uptake and metabolism (*exbD*, *hmuVST*, *fhuA2*), and genes encoding MDR efflux systems (*emrAB*).

The different comparisons performed in this study allowed the identification of 480 *S. meliloti* genes (205 up-regulated and 275 down-regulated) whose expression is altered specifically in response to myxobacterial predation (Supplementary Tables 7A, B). These defensome-specific genes belong to different functional categories, including many hypothetical genes. Nevertheless, the specific induction of several genes involved in cell wall and membrane biogenesis, as well as genes like *bacA*, *sodB*, or *irr*, suggests a role for cell envelope remodeling, transport of peptides, production of H_2_O_2_ and iron homeostasis in the adaptation/resistance response of *S. meliloti* cells against predation that awaits further experimentation.

## 4. Conclusion

**FIGURE 6:**
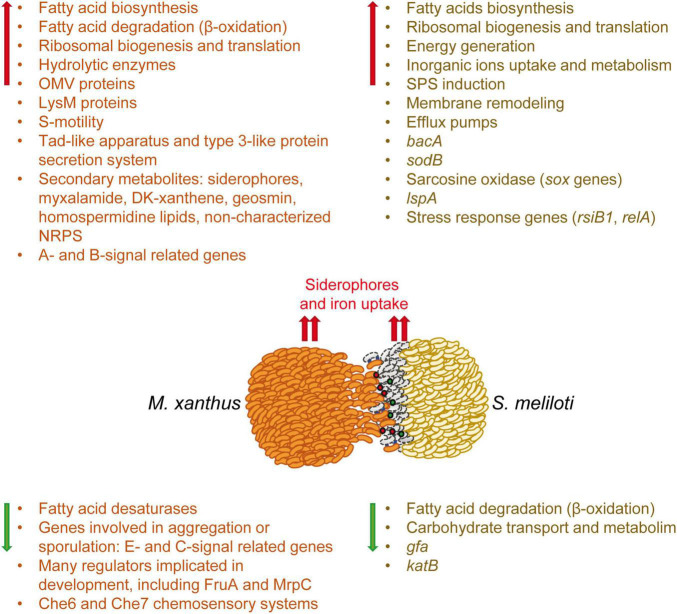
Schematic representation of the weapons used by the predator and the transcriptomic changes which occur in both the predator (predatosome) and the prey (defensome). Predatosome data have been compiled from Pérez et al. (2022). Red and green arrows represent up-regulation and down-regulation, respectively. Red and green balls represent extracellular weapons used by *Myxococcus xanthus*, such as hydrolytic enzymes and secondary metabolites, while blue lines represent contact dependent mechanisms. The dead prey cells at the interface are drawn as gray bacilli.

This work completes the study at the transcriptional level of the predator-prey interaction established between *M. xanthus* and *S. meliloti*, which was previously initiated with the analyses of the *M. xanthus* predatosome. Here, we have analyzed the defensome of *S. meliloti*, that is, the transcriptomic changes that the prey undergoes when facing the predator. The main transcriptomic changes observed in the predator (Pérez et al., 2022) and in the prey have been compiled in Figure 6. This analysis has revealed that the interaction with *M. xanthus* causes a massive remodeling of the *S. meliloti* transcriptome that affects more than a quarter of the genome, indicating a strong adaptation/defense response of the prey. However, it is difficult to know if the observed transcriptional changes are triggered upon the detection of *M. xanthus* cells or secreted factors, by lysed *S. meliloti* cells, or both. Further investigations might give clues to answer the question.

*Sinorhizobium meliloti* response involves the activation of mostly chromosomal-encoded functions, whereas the symbiotic plasmids have a minor contribution. During myxobacterial predation, *S. meliloti* up-regulates the expression of genes involved in protein synthesis and secretion, energy generation, fatty acid synthesis, and inorganic ion uptake and metabolism, while down-regulating genes required for FA degradation and carbohydrate transport and metabolism. This study highlights several significant changes that take place in the cell envelope of *S. meliloti* during myxobacterial predation. The up-regulation of many genes involved in the synthesis of different SPSs (EPS I, EPS II, KPS, PPP) suggest an increase in SPS production that could protect the cell by acting as a barrier against the action of hydrolytic enzymes and other antimicrobial compounds released by the predator. In addition to increased SPS production, transcriptomic changes detected in response to myxobacterial predation indicate the existence of modifications related to peptidoglycan and LPS, as well as significant membrane remodeling with the increased synthesis of different phospholipids and phosphorous-free membrane lipids. As previously observed in the predator, a remarkable up-regulation of iron-uptake mechanisms also takes place in *S. meliloti*, indicating that during the interaction, both the predator and the prey are experiencing iron starvation and need to compete for this essential nutrient.

Importantly, our findings have unveiled defensive mechanisms potentially used by *S. meliloti* during predatory attack. In addition to increased SPS production and modifications of the LPS that could protect rhizobial cells against membrane-damaging compounds produced by the predator, the activation of MDR efflux pumps and the broad-specificity peptide uptake transporter BacA could also contribute to defend *S. meliloti* from toxic compounds and membrane-damaging peptides potentially produced by *M. xanthus*. Additional defensive mechanisms include the increased expression of LspA to counteract the production of the antibiotic myxovirescin, and the production of formaldehyde and hydrogen peroxide that could have detrimental effects on the predator.

## Data availability statement

The datasets presented in this study can be found in online repositories. The names of the repository/repositories and accession number(s) can be found below: NCBI–PRJNA937699.

## Author contributions

MS and JP: substantial contributions to conception, design, analysis, interpretation of data, draft the manuscript and revising it critically for important intellectual content, and funding acquisition. JM-D: substantial contributions to conception, design and interpretation of the data, revising the manuscript critically for important intellectual content, and funding acquisition. FC-M: acquisition of data and revising the manuscript critically for important intellectual content. AM-M: acquisition of data, editing and revising the manuscript critically for important intellectual content, and funding acquisition. All authors contributed to the article and approved the submitted version.

## Footnotes

1
https://iant.toulouse.inra.fr/bacteria/annotation/cgi/rhime.cgi
