# Supplementary material for: Transcriptomic response of Sinorhizobium meliloti to the predatory attack of Myxococcus xanthus
Source: Front Microbiol. 2023 Jun 19;14:1213659. doi: 10.3389/fmicb.2023.1213659 (PMC10315480; doi:10.3389/fmicb.2023.1213659)
Supplement: Supplementary file 1 [file Data_Sheet_1.zip › Supplementary Figures S1-S5.pdf]

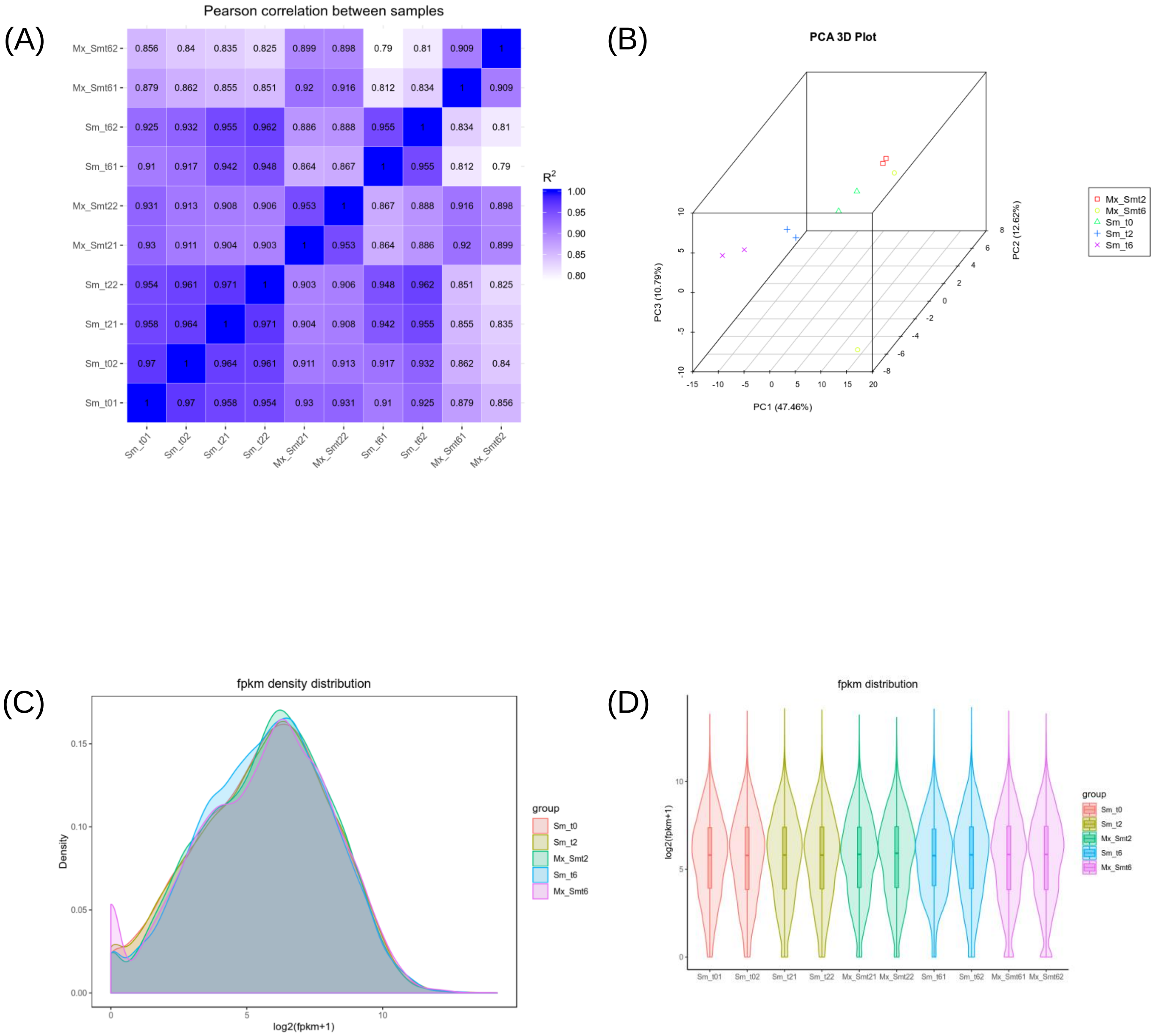

Figure S1. **Comparisons of the FPKM values and correlation between samples.** (**A)** Pearson correlation. t0, t2 and t6 means 0, 2 or 6 hours, respectively, of *S. meliloti* pure culture (Sm) or interaction with *M. xanthus* (Mx_Sm)*.* Two replicates were generated for the different conditions. (**B)** Principal component analysis (PCA) of RNA-seq. The R statistical language global gene was used to analyze the global expression data from each time point. Colored marks symbolize individual biological replicates harvested at the different conditions and times. **(C)** FPKM density distribution. The x-axis shows the log10 (FPKM+1) and the y-axis shows gene density. (**D)** FPKM violin plots. The x-axis shows the sample names and the y-axis shows the log10 (FPKM+1).

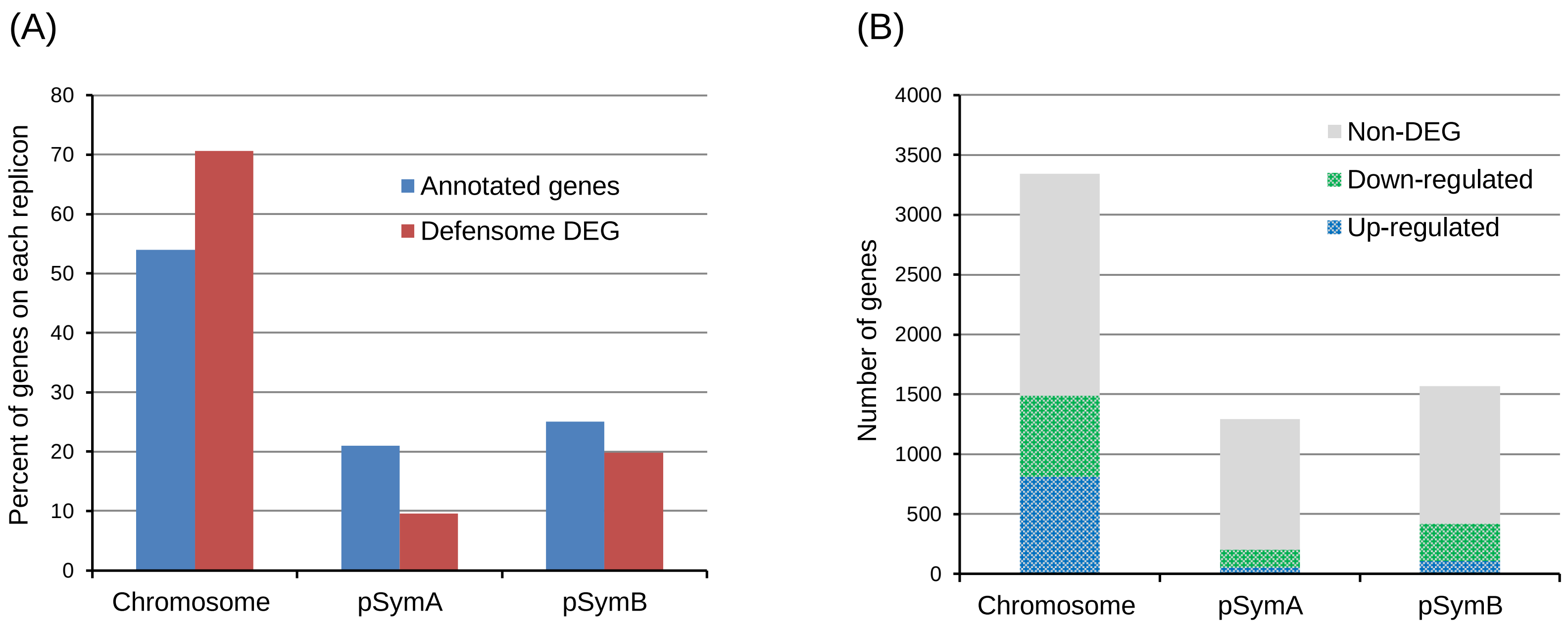

Figure S2. **Distribution of defensome genes on the *S. meliloti* replicons**. **(A)** The percent of *S. meliloti* genes located on each replicon is shown for the annotated genes (blue bars) and the genes with altered expression during predation by *M. xanthus* (red bars). **(B)** Number of differentially expressed genes in response to predation by *M. xanthus* on each replicon.

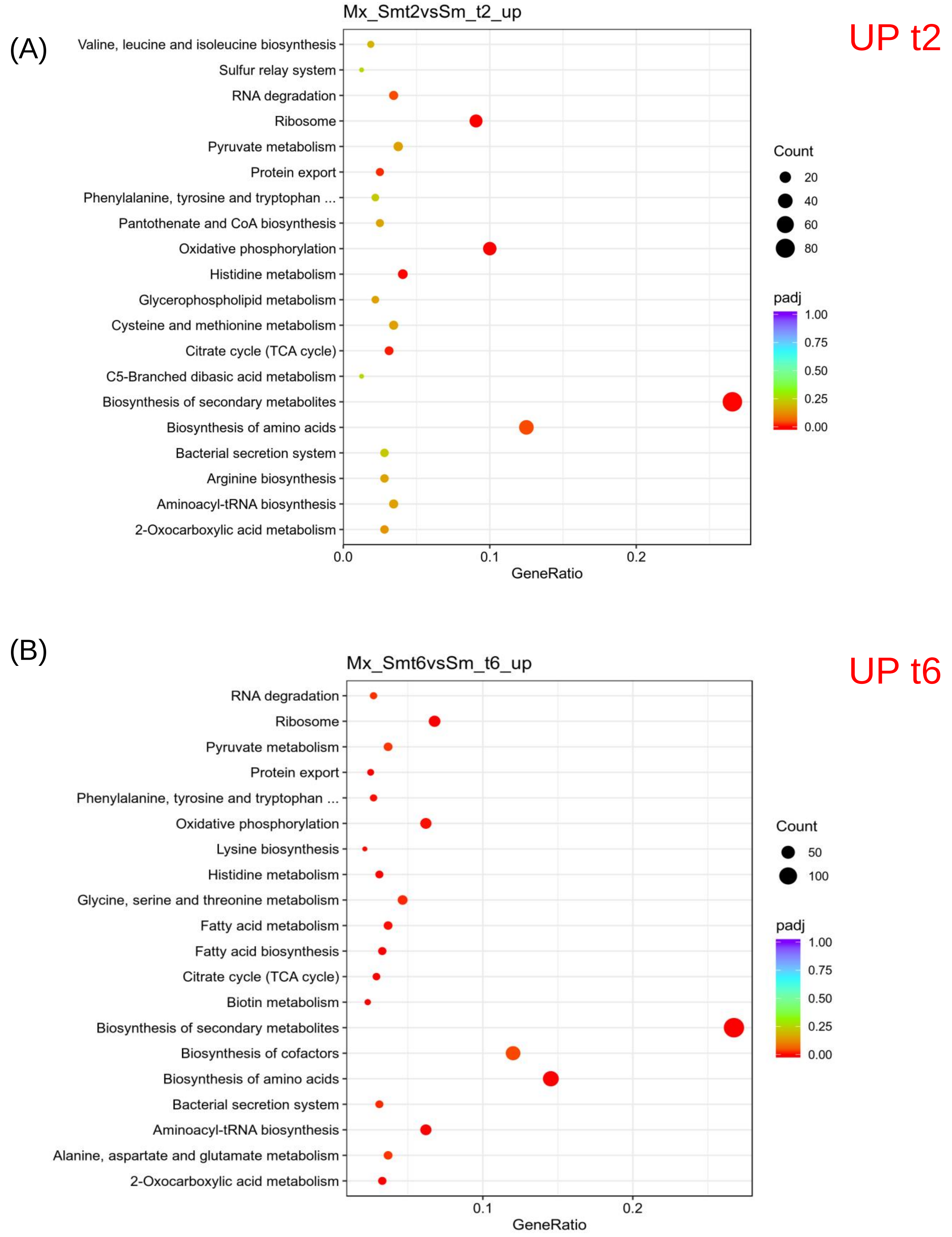

Figure S3A. **Enrichment of genes up-regulated in *S. meliloti* during predation by *M. xanthus* in significant KEGG pathways. (A)** Predation time 2 hours (t2): Mx_Smt2 vs Smt2. **(B)** predation time 6 hours (t6); Mx_Smt6 vs Smt6. The y-axis shows the KEGG pathway modules and the x-axis shows the ratio of differentially expressed genes to all genes concerned to this KEGG pathway module. See Table S1E and S1F.

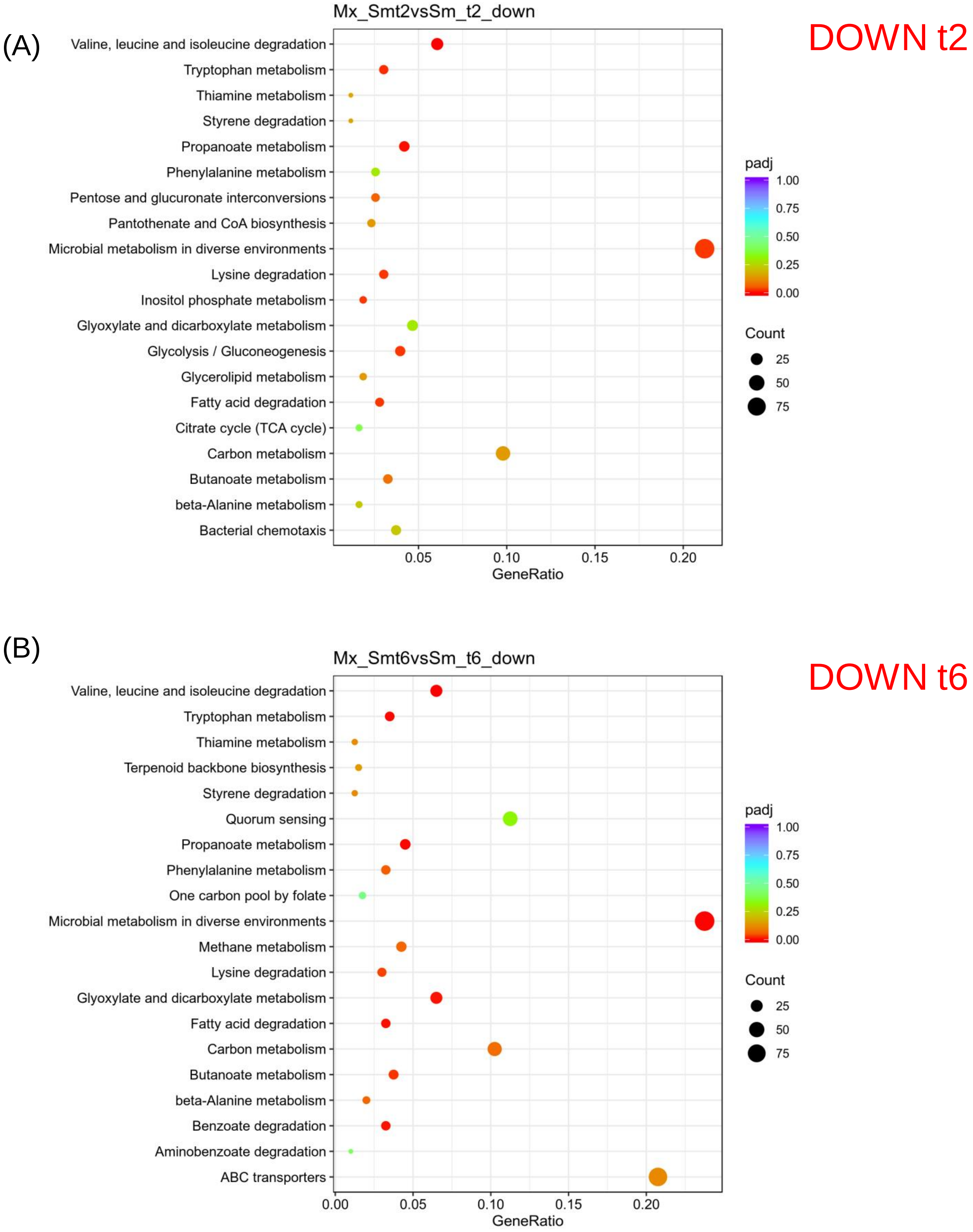

Figure S3B. **Enrichment of genes down-regulated in *S. melilloti* during predation by *M. xanthus* in significant KEGG pathways.** (**A**) Predation time 2 hours (t2): Mx_Smt2 vs Smt2 and (**B**) predation time 6 hours (t6); Mx_Smt6 vs Smt6. The y-axis shows the KEGG pathway modules and the x-axis shows the ratio of differentially expressed genes to all genes concerned to this KEGG pathway module. See Table S1E and S1F.

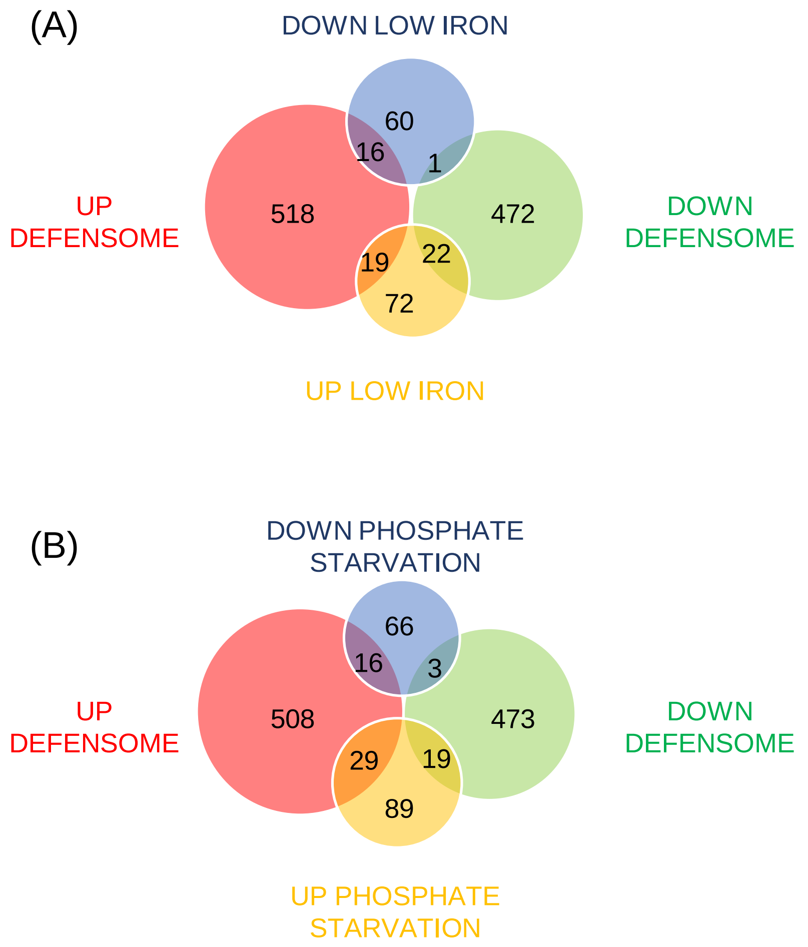

Figure S4. **Comparison of up- and down-regulated genes (|Log2 Fold Change|>1) in the defensome at 2 and/or 6 hours with the transcriptional response of *S. meliloti* to (A) iron starvation** (Chao et al., 2005) **and (B) phosphate starvation** (Krol and Becker, 2004). See Table S4A, S4B, S4C, and S4D.

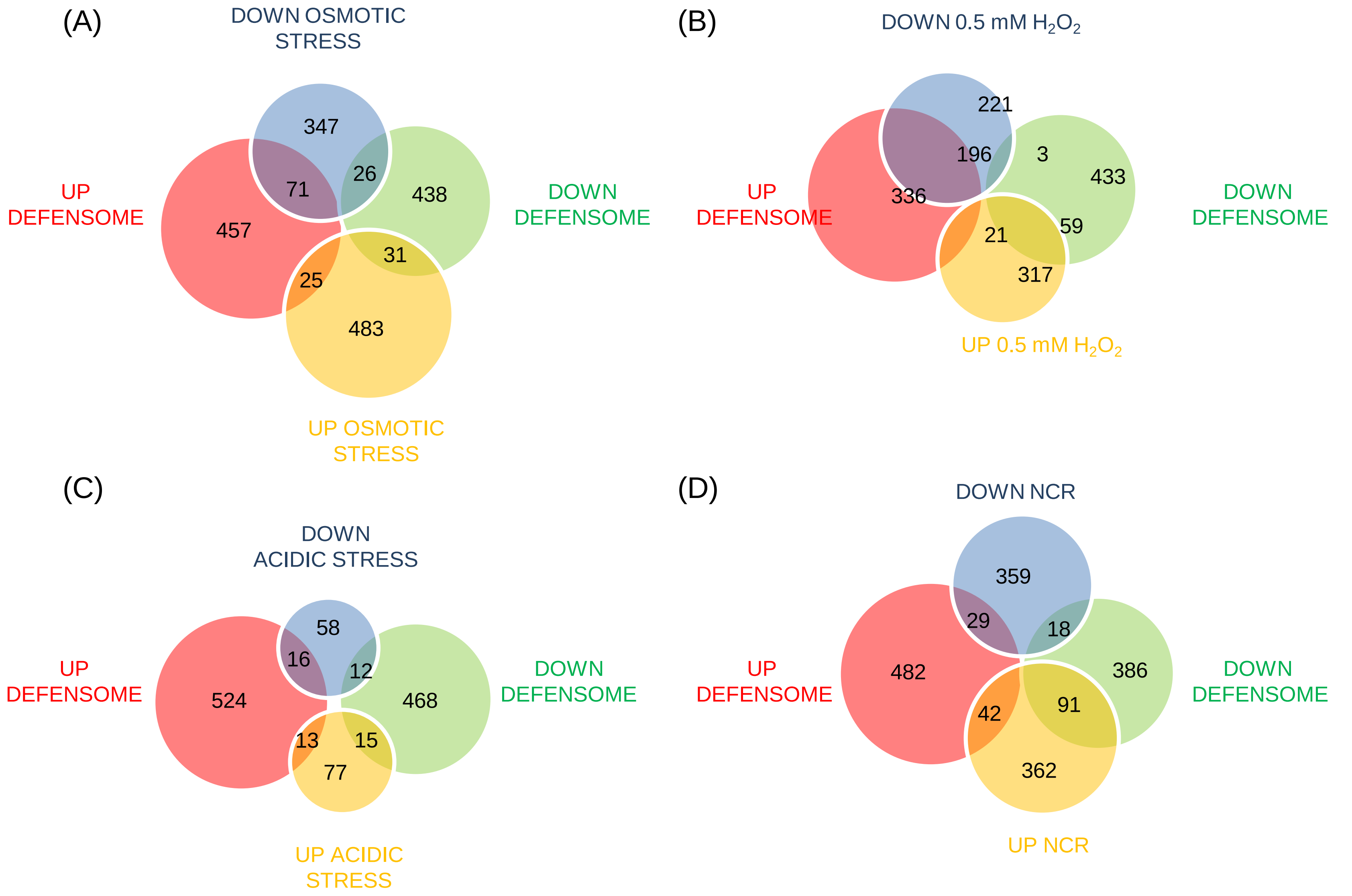

Figure S5. **Comparison of up- and down-regulated genes in the defensome (|Log2 Fold Change|>1) at 2 and/or 6 hours with the transcriptional response of *S. meliloti* to abiotic stresses. (A)** Osmilarity stress elicited by addition of either NaCl or sucrose (Domínguez-Ferreras *et al*., 2006). **(B)** Transcriptional response to H_2O_2 (0.5 mM) for *S. meliloti* wild-type strain compared to a mutant deficient in the key oxidative regulatory protein OxyR (Lehman and Long, 2018). **(C)** Acidic stress following a pH shift from pH 7.0 to pH 5.75 (Hellweg *et al*., 2009). **(D)** Transcriptional response of *S. meliloti* against nodule-specific cysteine-rich (NCR) peptides (Penterman *et al*., 2014). See Table S6A-H.
